# Exogenous trehalose improves growth under limiting nitrogen through upregulation of nitrogen metabolism

**DOI:** 10.1186/s12870-017-1207-z

**Published:** 2017-12-19

**Authors:** Yingchao Lin, Jie Zhang, Weichang Gao, Yi Chen, Hongxun Li, David W. Lawlor, Matthew J. Paul, Wenjie Pan

**Affiliations:** 1 Guizhou Academy of Tobacco Science, Guiyang, 550081 People’s Republic of China; 2 Upland Flue-Cured Tobacco Quality and Ecology Key Laboratory of China Tobacco, Guiyang, 550081 People’s Republic of China; 3 Formerly Rothamsted Research, Harpenden, Hertfordshire, AL5 2JQ UK; 4 Plant Science, Rothamsted Research, Harpenden, Hertfordshire, AL5 2JQ UK

**Keywords:** Trehalose, NO_3_^−^ assimilation, NH_4_^+^ assimilation, *Nicotiana tabacum*, Nitrogen

## Abstract

**Background:**

The trehalose (Tre) pathway has strong effects on growth and development in plants through regulation of carbon metabolism. Altering either Tre or trehalose 6-phosphate (T6P) can improve growth and productivity of plants as observed under different water availability. As yet, there are no reports of the effects of modification of Tre orT6P on plant performance under limiting nutrition.

**Results:**

Here we report that nitrogen (N) metabolism is positively affected by exogenous application of Tre in nitrogen-deficient growing conditions. Spraying foliage of tobacco (*Nicotiana tabacum*) with trehalose partially alleviated symptoms of nitrogen deficiency through upregulation of nitrate and ammonia assimilation and increasing activities of nitrate reductase (NR), glycolate oxidase (GO), glutamine synthetase (GS) and glutamine oxoglutarate aminotransferase (GOGAT) with concomitant changes in ammonium (NH_4_^+^) and nitrate (NO_3_^−^) concentrations, glutamine and amino acids. Chlorophyll and total nitrogen content of leaves and rates of photosynthesis were increased compared to nitrogen-deficient plants without applied Tre. Total plant biomass accumulation was also higher in Tre -fed nitrogen-deficient plants, with a smaller proportion of dry weight partitioned to roots, compared to nitrogen-deficient plants without applied Tre. Consistent with higher nitrogen assimilation and growth, Tre application reduced foliar starch. Minimal effects of Tre feeding were observed on nitrogen-sufficient plants.

**Conclusions:**

The data show, for the first time, significant stimulatory effects of exogenous Tre on nitrogen metabolism and growth in plants growing under deficient nitrogen. Under such adverse conditions metabolism is regulated for survival rather than productivity. Application of Tre can alter this regulation towards maintenance of productive functions under low nitrogen. This has implications for considering approaches to modifying the Tre pathway for to improve crop nitrogen-use efficiency and production.

## Background

The trehalose pathway in plants has emerged over 20 years as an important regulatory system linking sucrose supply with growth and development [1, 2]. Trehalose is synthesized in plants in a low flux pathway exclusively via the intermediate trehalose 6-phosphate (T6P). Modification of the pathway in transgenic plants and through exogenous application of Tre or T6P has large effects on growth and development [3–6]. Overall a consensus is emerging that the regulatory function of the Tre pathway in plants is to coordinate carbon supply, particularly sucrose, with metabolism, growth and development, so that appropriate physiological responses are elicited in the light of prevailing carbon resource availability [7]. As shown by recent progress through genetic modification, marker-assisted selection and chemical intervention [6, 8, 9], the Tre pathway offers considerable opportunity in crop improvement, the challenge being to target such a powerful regulatory mechanism for different crops and conditions.

Trehalose is easily and cheaply available and, when applied exogenously, is readily taken up by plants. It has been used as a tool to perturb plant processes to better understand the mechanisms that determine plant growth and yield. Feeding Tre in 100 mM concentrations strongly inhibits growth [5, 10] with large effects on starch metabolism and ADP-glucose pyrophosphorylase transcription and redox activation [11] associated with starch accumulation in source tissues [12]. The exact mechanistic basis of the strong inhibition of growth induced by Tre feeding is not clear but may relate to perturbation of the normal functioning of the T6P/SnRK1 signaling pathway [5]. Bae et al. [13] showed that feeding 30 mM Tre affected many transcripts in Arabidopsis seedlings including transcription factors, defense- and stress-related transcripts and transcripts involved in metabolism. Exogenous Tre feeding in low millimolar concentrations to salt stressed *Arabidopsis* [14] and *Catharanthus* [15] showed that salt resistance was improved through regulation of plant redox state, cell death, ion distribution and osmotic adjustment. Furthermore, applying Tre directly to wheat leaves by foliar spraying induced the activities of various defense-response proteins and conferred resistance to powdery mildew [16].

Nitrogen metabolism is closely integrated with carbon metabolism in all areas of plant function, including photosynthesis where the photorespiratory nitrogen cycle results in considerable turn-over of amino acids and of total nitrogen [17]. Genetic modification of T6P impacts photosynthesis and investment in photosynthetic machinery [4] and up-regulates amino acid metabolism [18, 19]. Modification of the Tre pathway potentially, therefore, provides a means to modify nitrogen metabolism and improve nitrogen-use efficiency in crop production.

In the current study, the effect of a Tre foliar spray on the performance of *Nicotiana tabacum* grown under nitrogen deficiency was determined to test the hypothesis and proof of concept that modification of Tre levels in plants could be a means to improve performance under limiting nitrogen. Given that flux through the Tre pathway and elevated levels of both T6P and Tre are associated with biosynthetic processes including upregulation of transcripts and enzyme activities of nitrogen metabolism [13, 18, 19] we wished to determine if Tre-induced upregulation of nitrogen metabolism would improve plant performance under limiting nitrogen. Strikingly, it was found that Tre application under low nitrogen increased assimilation of both nitrate and ammonium associated with increased activities of NR and enzymes of photorespiratory ammonium re-assimilation and altered accumulation of amino acids. This stimulation was correlated with improved photosynthesis, growth and final biomass of plants. The study provides further insight into the range of processes affected by the Tre pathway in plants and for the first time shows that under nitrogen-deficient conditions Tre can induce strong up-regulation of nitrogen assimilatory capacity improving plant performance.

## Results

### Effects of exogenous Tre on chlorophyll concentration under contrasted N

**Fig. 1:**
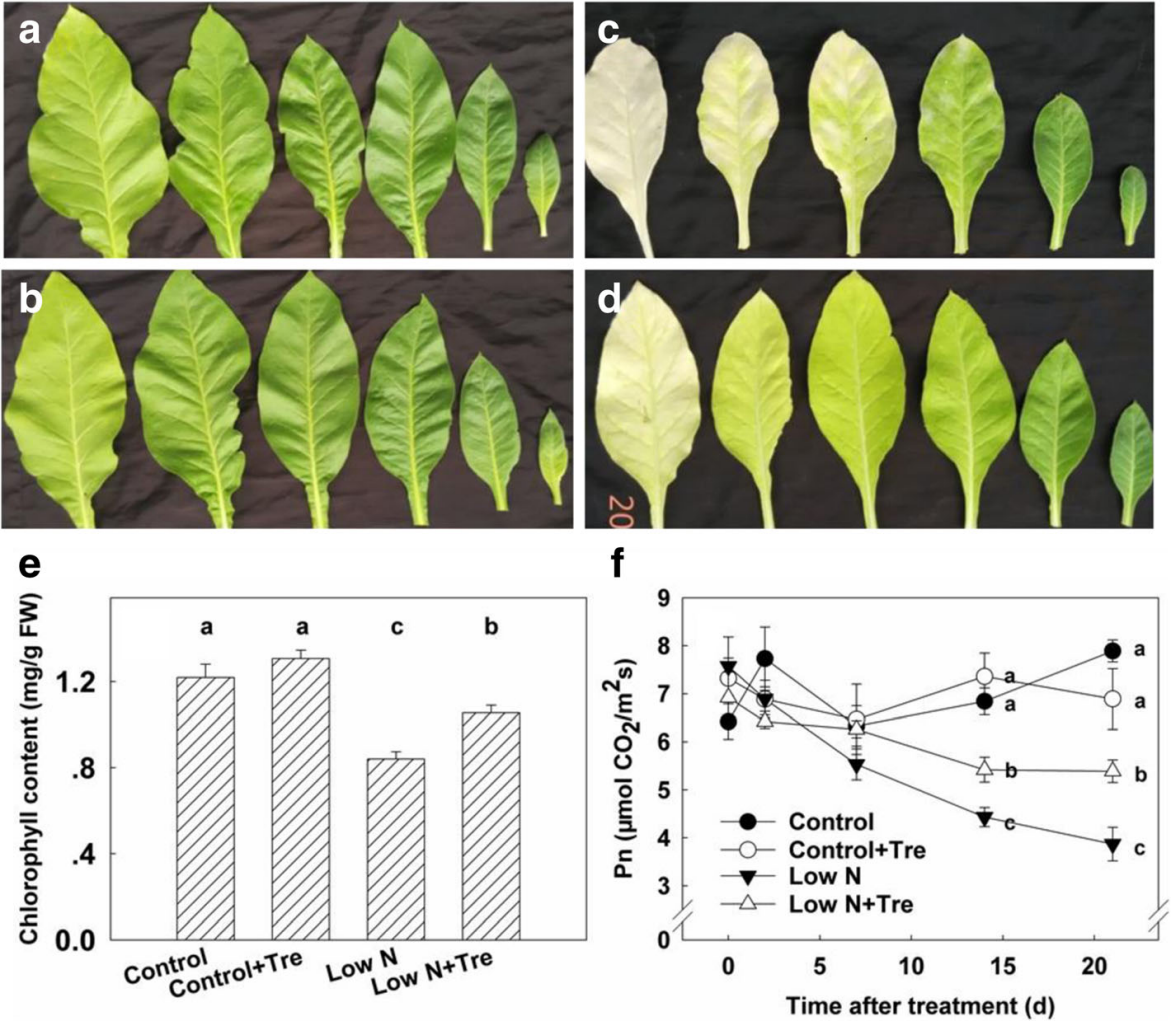
Leaves of tobacco seedlings without (**a** and **c**) or with (**b** and **d**) exogenous trehalose under control (**a** and **b**) or nitrogen deficient (**c** and **d**) conditions. Chlorophyll content (**e**) and photosynthesis rate (**f**) of the youngest fully expanded leaf. Pictures and data were obtained from seedlings (45 days old) after treatment for 15 days as indicated except (**d**). Values represent the mean ± SE of 5 individual plants. Within each set of experiments, the different letters indicate significant differences (*P* < 0.05)

To investigate the effects of Tre on nitrogen metabolism and photosynthesis exogenous Tre was applied to nitrogen-deficient and nitrogen-sufficient plants for 21 days. During this period, the chlorophyll content of leaves grown with deficient nitrogen decreased greatly compared to those grown at sufficient nitrogen (Fig. 1a–e), particularly for older leaves below the third leaf from top. However, exogenous Tre slowed loss of chlorophyll from leaves under low nitrogen (Fig. 1d and e) but had only minor visible effects under sufficient nitrogen (Fig. 1b and e). Net photosynthesis rates of the third leaves were also determined. Rates were not significantly affected after low nitrogen for 7 days, but decreased significantly subsequently compared to those of nitrogen-sufficient plants. However, exogenously applied Tre significantly improved photosynthesis for nitrogen-deficient plants (Fig. 1f).

### Effects of exogenous Tre on endogenous Tre and T6P content under contrasted N

**Fig. 2:**
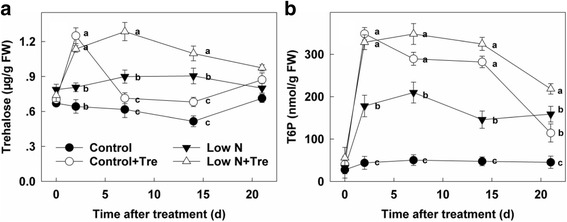
The contents of trehalose (**a**) and T6P (**b**) in the youngest fully expanded leaf after different treatment for 0, 2, 7, 14 and 21 days as indicated. Values represent the mean ± SE of 5 individual plants. Within each set of experiments, the different letters indicate significant differences (*P* < 0.05)

As the results showed, contents of endogenous Tre in plants grown with deficient N without application of Tre are higher than those of the control with sufficient nitrogen. Moreover, exogenous application of Tre further increased endogenous Tre in both nitrogen treatments while sudden decline of the peak after treatment in control plant was observed (Fig. 2a).

Trehalose 6-phosphate, an intermediate in Tre synthesis, is a signal molecule involved in many developmental processes. To investigate Tre metabolism under nitrogen deficient conditions, we measured T6P content in fully expanded leaves of seedlings. Under limiting nitrogen the content of T6P increased significantly compared to that of control seedlings: application of exogenous Tre further induced large increases in T6P content. Moreover, application of exogenous Tre also significantly increased T6P content in the control seedlings, and the content of T6P was higher than those of low N plants until 21 d after treatment (Fig. 2b).

### Effects of exogenous Tre on nitrate, ammonium, total nitrogen contents and NR activity under contrasting N

**Fig. 3:**
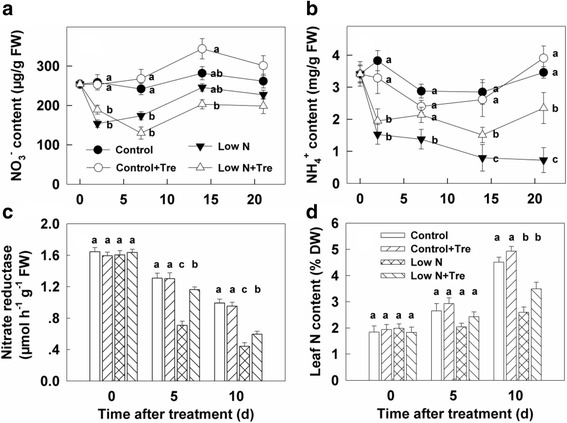
The contents of NO_3_^−^ (**a**) and NH_4_^+^ (**b**), nitrate reductase activity (**c**) and leaf total nitrogen content (**d**) in the youngest fully expanded leaf after different treatment for different time as indicated. Values represent the mean ± SE of 5 individual plants. Within each set of experiments, the different letters indicate significant differences (*P* < 0.05)

Both the nitrate and ammonium decreased in plants grown under low nitrogen compared to sufficient nitrogen. Exogenous Tre increased ammonium but decreased nitrate under low nitrogen conditions. Under sufficient nitrogen, although with less significant differences, exogenous Tre tended to increase the nitrate and caused an effect on ammonium (Fig. 3a and b). Increased NR activity and higher leaf total nitrogen content under low nitrogen resulted from Tre feeding, whereas exogenous Tre had only small effects on these parameters under nitrogen-sufficient conditions (Fig. 3c and d).

### Effects of exogenous Tre on glycollate and GO under contrasted N

**Fig. 4:**
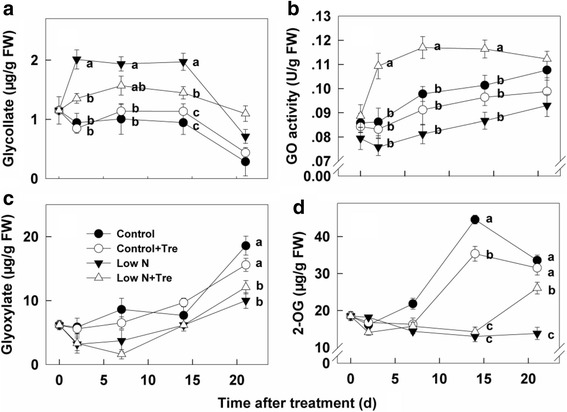
The contents of Glycollate (**a**), Glyoxylate (**c**), 2-OG (**d**) and activity of GO (**b**) in the youngest fully expanded leaf after different treatment for 0, 2, 7, 14 and 21 days. Values represent the mean ± SE of 5 individual plants. Within each set of experiments, the different letters indicate significant differences (*P* < 0.05)

The impact of exogenous Tre on photorespiratory metabolites was determined. Accumulation of glycollate increased substantially under low nitrogen compared to sufficient nitrogen, but was decreased by exogenous Tre at low nitrogen (Fig. 4a). Activities of GO under low nitrogen conditions decreased compared to those with adequate nitrogen. Application of exogenous Tre under low nitrogen conditions increased GO activities greatly compared to controls (Fig. 4b). The content of glyoxylate decreased after 7 days of low nitrogen treatment compared to sufficient nitrogen, but there were no notable effects with exogenous Tre (Fig. 4c). The content of 2-oxoglutarate (2-OG) decreased slightly with applied Tre at sufficient nitrogen but increased at day 21 under nitrogen-deficient conditions. Concentrations were decreased compared to high nitrogen between days 14–21 (Fig. 4d).

### Effects of exogenous Tre on Gln and glutamate (Glu) concentrations and GS and GOGAT activities under contrasted N

**Fig. 5:**
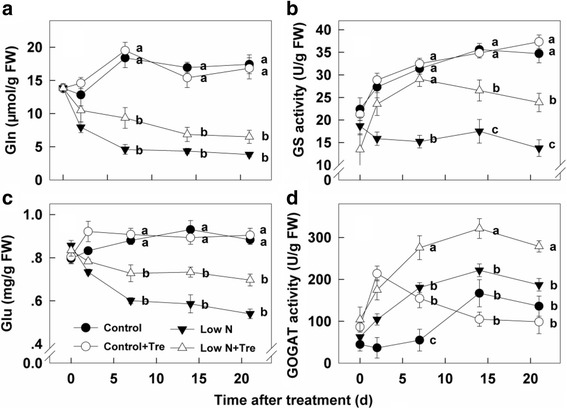
The contents of Gln (**a**), Glu (**c**) and activities of GS (**b**), GOGAT (**d**) in the youngest fully expanded leaf after different treatment for 0, 2, 7, 14 and 21 days. Values represent the mean ± SE of 5 individual plants. Within each set of experiments, the different letters indicate significant differences (*P* < 0.05)

Both Gln and Glu decreased substantially at low nitrogen compared to sufficient nitrogen. Exogenous Tre at sufficient nitrogen did not increase Gln or Glu but did at low nitrogen (Fig. 5a and c). GS activity increased under nitrogen sufficient conditions but not at deficient nitrogen. Tre application increased activity with deficient nitrogen, but not sufficient nitrogen (Fig. 5b). Application of Tre increased GOGAT activity strongly initially under nitrogen-sufficient conditions and then decreased gradually to those of control without exogenous Tre. Nitrogen deficiency increased GOGAT activity compared to nitrogen-sufficient plants and application of exogenous Tre increased it further (Fig. 5d).

### Effects of exogenous Tre on amino acids concentrations under contrasted N

**Fig. 6:**
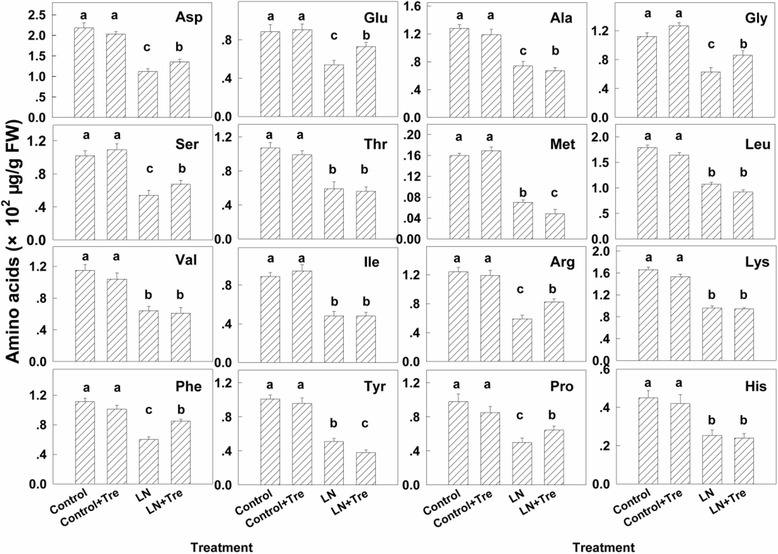
The contents of free amino acids in the youngest fully expanded leaf after different treatment for 21 days. Values represent the mean ± SE of 5 individual plants and the different letters indicate significant differences (*P* < 0.05)

To investigate the effects of application of exogenous Tre for 21 days, compared to the control, on nitrogen metabolism and utilization after long-term growth of the plants with N deficiency or sufficiency, the content of amino acids in the youngest fully expanded leaves were analyzed. Generally, the content of amino acids was nearly halved under low nitrogen compared to nitrogen-sufficient conditions. Exogenous Tre significantly increased the contents of aspartate (Asp), glutamate (Glu), glycine (Gly), serine (Ser), arginine (Arg), phenylalanine (Phe) and proline (Pro) but decreased those of alanine (Ala), methionine (Met), leucine (Leu) and Tyrosine (Tyr) significantly under low nitrogen but had minimal effects in nitrogen-sufficient plants (Fig. 6).

### Effects of exogenous Tre on Tre pathway genes expression under contrasted N

**Fig. 7:**
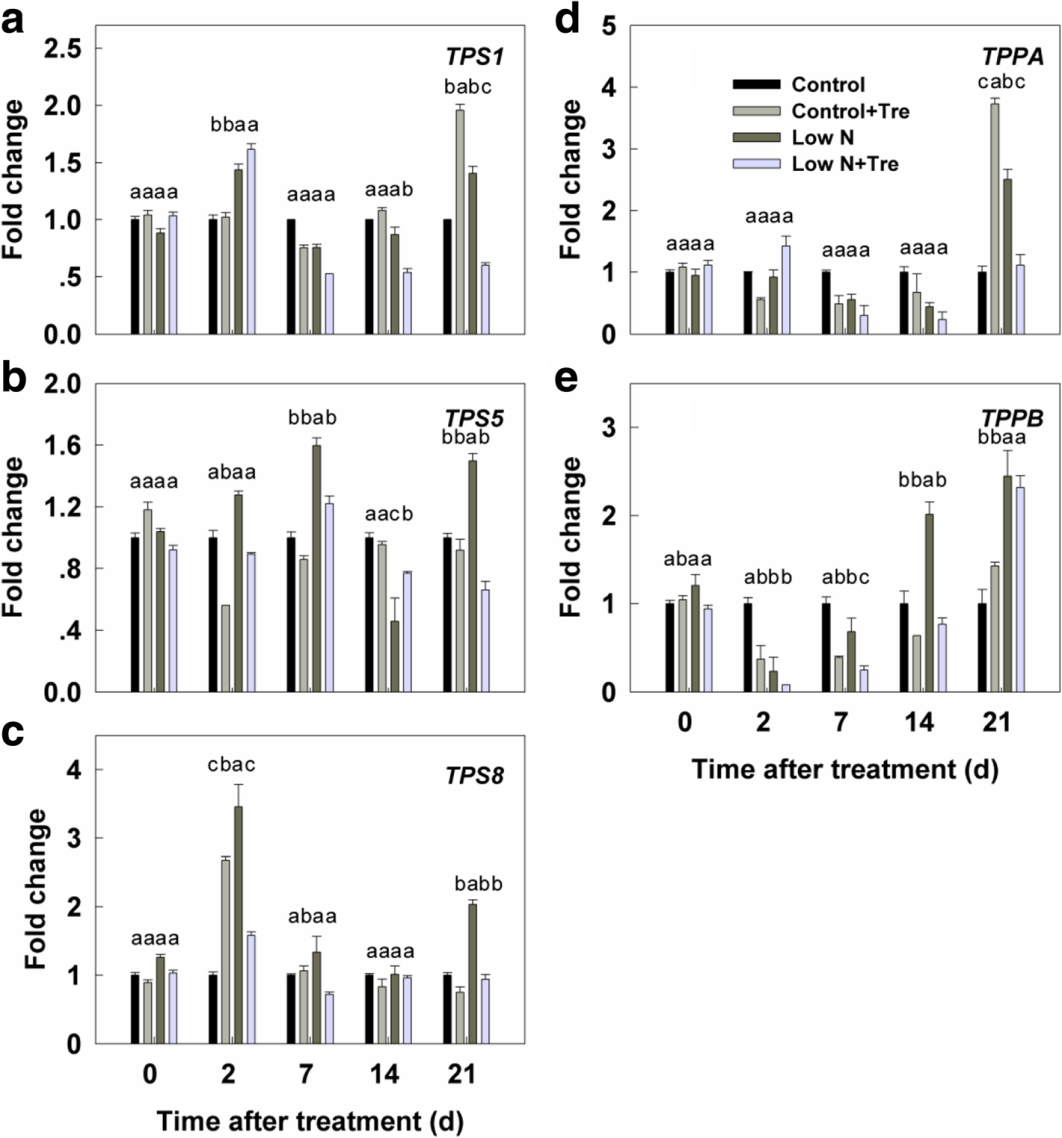
Relative expression of trehalose phosphate synthase genes (**a**, **b** and **c**) and trehalose phosphate phosphatase genes (**d** and **e**) in the youngest fully expanded leaf determined by qRT-PCR in response to low nitrogen and exogenous trehalose. Values represent the mean ± SE of 5 individual plants. Within each set of experiments, the different letters indicate significant differences (*P* < 0.05)

To examine how the Tre pathway responded to the treatments, transcript abundances of *TPS1*, *TPPA* and *TPPB* as representative genes of the pathway known to be catalytically active [21] were determined. Quantitative reverse transcription (qRT)-PCR analysis of the genes involved in T6P biosynthesis and breakdown showed a relationship between T6P and external nitrogen supply but even larger effects of exogenous Tre [21]. Application of exogenous Tre under low nitrogen drastically upregulated *TPS1* expression, which then gradually decreased (Fig. 7a). Two Tre phosphate phosphatase (TPP) genes, *TPPA* and *TPPB* were both suppressed by nitrogen deficiency as well as by application of exogenous Tre, particularly under nitrogen deficiency, however, both were generally up regulated by exogenous Tre under nitrogen-deficient conditions in the longer term (21 days, Fig. 7d and e).

In growing tissues T6P is an inhibitor of SnRK1 which acts as transcriptional integrator in response to carbon and energy supply, thus transcript abundances of SnRK1 marker genes including *TPS5* and *TPS8* were analyzed to verify the regulation of T6P. Tre phosphate synthase (TPS) genes *TPS5* and *TPS8* were generally upregulated by nitrogen deficiency after 2 days, but were little altered by exogenous Tre under nitrogen-deficient conditions (Fig. 7b and c).

### Effects of exogenous Tre on sugars and starch under contrasted N

**Fig. 8:**
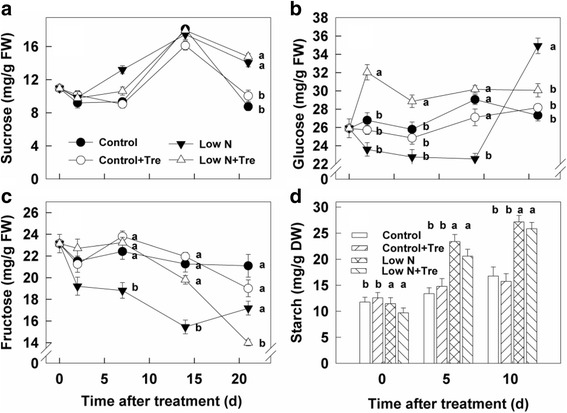
The contents of sucrose (**a**), glucose (**b**), fructose (**c**) and starch (**d**) in the youngest fully expanded leaf under sufficienct and deficient nitrogen with and without exogenous trehalose. Values represent the mean ± SE of 5 individual plants. Within each set of experiments, the different letters indicate significant differences (*P* < 0.05)

Amounts of sucrose in leaves were little affected by nitrogen or Tre treatment (Fig. 8a). Glucose concentrations were generally decreased under nitrogen deficiency compared to nitrogen sufficiency, but elevated by Tre at low nitrogen only (Fig. 8b). Fructose was decreased by low nitrogen but again increased by exogenous Tre only at low nitrogen (Fig. 8c). Starch was increased substantially by low compared to high nitrogen and decreased by exogenous Tre under low nitrogen but not at high nitrogen (Fig. 8d).

### Effects of exogenous Tre on plant growth under contrasted N

**Fig. 9:**
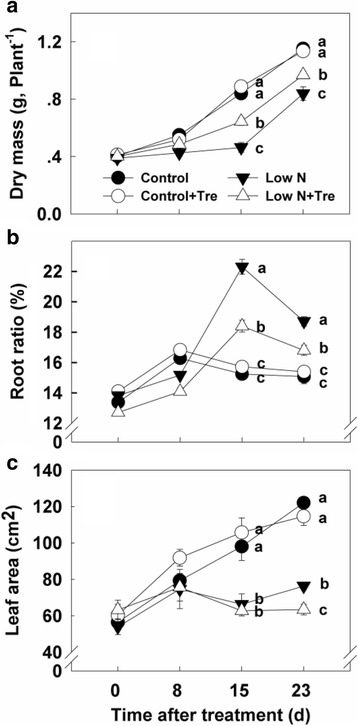
Effects of exogenous trehalose and nitrogen supply on plant dry mass (**a**), root to total dry matter ratio (**b**) and leaf area (**c**). Values represent the mean ± SE of 5 individual plants. Within each set of experiments, the different letters indicate significant differences (*P* < 0.05)

Exogenous application of trehalose did not affect growth and biomass production of plants grown with sufficient nitrogen (Fig. 9a-c). However, in nitrogen-deficient plants Tre increased growth and final dry weight and decreased the overall proportion of root to shoot biomass. At deficient, compared to abundant nitrogen there was more root throughout and less leaf area at day 23 (Fig. 9b, c).

## Discussion

The strong regulatory role of the Tre pathway in plants is well known [20], but here for the first time we show that exogenous Tre can improve plant growth and performance under limiting nitrogen growing conditions associated with strong upregulation of nitrate and ammonium assimilation.

Low nitrogen without any exogenous Tre treatment resulted in more endogenous T6P and Tre in tobacco leaves (Fig. 2) suggesting that flux through the pathway is increased in low nitrogen. This accords with previous observations [21] of elevated T6P in nitrogen-deficient seedlings and the correlation with increased sucrose. In the current study, however, no increase in sucrose was observed suggesting the possibility that nitrogen itself could exert specific regulation of the Tre pathway flux independently of sucrose. This aspect of the interaction between nitrogen deficiency and direct stimulation of the Tre pathway requires further investigation. Increased endogenous Tre under nitrogen deficiency was not associated with increased expression of transcripts of *TPS1*, the main catalytic TPS that would explain the increase (Fig. 7). TPPA and TPPB were not obviously increased either (Fig. 7d, e). Post-translational modifications of TPS1 or TPPA and TPPB and changed activities of trehalase may have contributed to altered Tre levels. Alternatively elevated trehalose and T6P were simply derived from the exogenous trehalose.

Exogenous Tre produced striking effects on activities of enzymes involved in nitrogen assimilation – NR, GO, GS and GOGAT (Figs. 3c, 4b, 5b, d). With the exception of GOGAT during the early part of the experiment (Fig. 5d) the increase in enzyme activities were confined to plants grown at low nitrogen. There were minimal effects on overall nitrogen, amino acid content or growth and performance through Tre feeding at sufficient nitrogen, therefore there is no indication that increased GOGAT activity during the early period at sufficient nitrogen improved plant performance under these conditions. Recently, it was shown that elevated T6P resulted in increased activity of NR through protein phosphorylation [19] implicating T6P in coordinating carbon and nitrogen metabolism in plants. An increased T6P content, as well as NR activity, after application of exogenous Tre to low nitrogen plants (Fig. 2b and 3c) were also observed in the present study, indicating that T6P is directly related to nitrogen metabolism under limiting nitrogen growing conditions. Whilst we have no direct proof in this study that T6P is the causal factor of increased NR activity we can show a potentially more widespread role for the Tre pathway beyond NR in the up-regulation of both nitrate assimilation and ammonium assimilation under limiting nitrate. Significantly, this resulted in a number of physiological changes and improvements in plant performance. Firstly, increased enzyme activities impacted on the intermediates of nitrate assimilation and ammonium assimilation. Nitrate concentration was decreased by Tre treatment at low nitrogen and ammonium was increased (Fig. 3a, b) consistent with the changes in enzyme activities observed. There was also a decrease in glycollate (Fig. 4a), a major intermediate in photorespiration. Overall the data are consistent with a perturbation of photorespiratory nitrogen assimilation due to the application of Tre to nitrogen-deficient plants.

Improved nitrate and photorespiratory nitrogen assimilation with application of Tre at low nitrogen supply increased leaf nitrogen (Fig. 3d) and several amino acids, including glutamate and glutamine (Fig. 5a, c; Fig. 6). Consistent with higher leaf nitrogen content, chlorophyll content and rates of photosynthesis were also greater (Fig. 1a-f). These changes resulted in improved overall biomass in plants growing at low nitrogen (Fig. 9a-c). However, during early stage of the experiment, the effects of Tre on growth under low nitrogen conditions were minor as there is no effect on leaf area (Fig. 9c). Consistent with more growth at low nitrogen due to Tre application there was a trend to lower starch (Fig. 8d). Roots are the principal site of nutrition and water uptake. Plants grown on low nitrogen generally have a larger root/shoot ratio than those grown with adequate nitrogen: this is considered to enhance exploitation of nitrogen in the soil. Interestingly, there was a reduction in allocation of biomass to roots in Tre-treated plants at low nitrogen (Fig. 9b). This suggests changes in regulatory mechanisms that determine shoot-root allocation possibly caused by an improved supply of nitrogen metabolites arising from increased nitrate and ammonium assimilatory enzymes. Amino acids can inhibit root growth [22] and many were elevated in this study. Glu has also been proposed as a regulatory molecule of root: shoot ratio [23].

Two main conclusions arise from the work. Firstly, exogenous Tre increases nitrogen assimilation, nitrogen concentration and growth by reversing the decreases in enzymes of nitrate and ammonium assimilation associated with nitrogen deficiency. Here we show that by decreasing the extent of the down-regulation of nitrogen metabolism under nitrogen deficiency, it is possible to increase productivity. Down regulation of nitrogen assimilation at low nitrogen is an adaption for survival. For agricultural systems, physiological processes in crops may still have too much of a legacy for survival rather than maximal productivity. As with the abortion of female reproductive structures under drought [9], the plant regulates responses to abiotic stress to ensure survival, not for maximal productivity. De-regulation of the adaptive responses to stress may improve productivity in marginal conditions such as drought [9] and under nitrogen deficiency, as shown in this study. Hence, for crops, increasing nitrogen metabolism capacity of younger organs such as those producing yield components under marginal nitrogen supply, may have utility in improving yields. A second major conclusion, arising from the observation that endogenous Tre is increased by low nitrogen, is that the Tre pathway may be involved in the regulation of nitrogen metabolic responses to nitrogen deficiency. By perturbing this regulation by further daily application of 8 mM Tre, large effects on nitrogen metabolism beyond effects on NR already observed through changes in T6P [19] are produced. Potentially therefore targeting the Tre pathway could be a means to upregulate nitrogen assimilatory metabolism to improve crop growth under limiting nitrogen conditions and potentially to improve nitrogen use efficiency. Daily treatment with Tre is not practicable in the agricultural environment, but does point towards the potential of this pathway for targeting through other approaches e.g. marker assisted selection already used to improve germination under flooding [8], genetic modification which has improved tolerance to drought at flowering [9] and T6P precursors which improved grain starch and grain size and yield and recovery from drought [6]. Further potential agricultural improvement of crop plants may come from modifying the Tre pathway targeting nitrogen metabolism and nitrogen use efficiency.

## Conclusions

The present study provides new insights into the involvement of Tre in regulation nitrogen assimilation under nitrogen use efficiency conditions. Endogenous Tre is increased by low nitrogen in tobacco plants, suggesting that Tre plays a role in adjustment of metabolism to deficient N conditions. Also, application of exogenous Tre increased plants growth by reversing the decreases in enzymes of nitrate and ammonium assimilation associated with nitrogen deficiency, indicating that the Tre pathway may be involved in the regulation of nitrogen responses to nitrogen deficiency. This has implications for considering approaches to modifying the Tre pathway to upregulate nitrogen assimilatory metabolism to improve crop growth under limiting nitrogen conditions and potentially to improve nitrogen use efficiency.

## Methods

### Plant material and growth conditions

Seeds of *Nicotiana tabacum* L. cv. K326 were sown in a glasshouse individually in plastic pots (4 × 4 × 5 cm) filled with organic soil composed of peat, humus and vermiculite, pH 5.0–6.8, watered regularly under a 12-h photoperiod at temperatures ranging from 19 °C (night) to 28 °C (day), average photosynthetic photon flux density of 600 μmol (photon) m^−2^ s^−1^ and relative humidity 80–85%. After 45 days, seedlings were transferred to plastic pots (diameter 15 cm; height 13 cm) filled with perlite and irrigated with Hoagland’s solution (previously found to be suitable for growing tobacco) containing either 1 mM NO_3_^−^ (low or deficient nitrogen) or 7.5 mM NO_3_^−^ (high or sufficient nitrogen) as described by Kavanová et al. [24]. The composition of the nutrient solution was: (1) high nitrogen: macronutrients: 2.5 mM Ca(NO_3_)_2_, 2.5 mM KNO_3_, 1 mM MgSO_4_, 0.18 mM KH_2_PO_4_, 0.21 mM K_2_HPO_4_, 0.5 mM NaCl, 0.4 mM KCl, 0.4 mM CaCl_2_; micronutrients: 125 μM Fe–ethylenediaminetetraacetic acid, 46 μM H_3_BO_3_, 9 μM MnSO_4_, 1 μM ZnSO_4_, 0.3 μM CuSO_4_, 0.1 μM Na2MoO_4_. (2) Low nitrogen: macronutrients: 1 mM KNO_3_, 1 mM MgSO_4_, 0.18 mM KH_2_PO_4_, 0.21 mM K_2_HPO_4_, 0.5 mM NaCl, 0.7 mM K_2_SO_4_, 2 mM CaCl_2_; micronutrients were the same as in high-nitrogen plants.

Half of the plants in the high nitrogen and half in the low nitrogen were treated by spraying 5 ml of 8 mM Tre on all the leaves on each plant with a manual pump early in the morning every day for 21 days. Control plants were sprayed with the same volume of pure water. Water was chosen as the Control treatment for Tre because it was considered not to introduce potential complications, which a different compound, such as sorbitol, might have done. The concentration of Tre applied was small, so its effects as an osmoticum and as an additional carbon source were negligible. Even if another compound was used, water would have had to be used as control. The experiment was laid out in a completely randomized design with 20 plants per treatment.

### Photosynthesis and chlorophyll content determination

Measurements of net photosynthetic rate (Pn) were made on the youngest fully expanded leaf using an LI-6400XT Portable Photosynthesis System (LI-COR, Lincoln, Nebraska USA). The light saturating photosynthetic rate was measured at a CO_2_ concentration of 400 μmol mol^−1^, 25 °C, relative humidity 80% and photon flux of 600 μmol (photon) m^−2^ s^−1^. Chlorophyll was extracted from freshly harvested fully expanded leaves using 80% acetone and analyzed according to [25].

### Trehalose and T6P determinations

For Tre extraction, 0.1 g of fresh leaf (youngest fully expanded leaf) was ground in liquid nitrogen and suspended in 2 mL of boiling ethanol according to El-Bashiti et al. [26]. Ethanol was then evaporated and the residue dissolved in 5 ml of the mobile phase (5 mM H_2_SO_4_) of the HPLC (LKB, BROMMA, 2150 HPLC pump). This solution was then centrifuged at 10,000 g for 10 min, filtered through 0.2 μm Millipore filter. Then the extract was incubated in boiling water for 1 h. Samples of this extract was analyzed using a monosaccharide column (Phenomenex, REZEX CAL, 300 mm × 7.8 mm, S/No. 40450) at flow rate of 0.5 ml min^−1^ and detected by refractory index detector (Knauer, differential-refractometer). Trehalose content was determined by comparing its chromatogram with that of different concentrations of commercial Tre (Sigma-Aldrich, Cat. No. T5251).

T6P were extracted from plant material as described in Delatte et al. [5]. Briefly, frozen tobacco material was ground and the powder and then extracted with a chloroform/MeCN mixture by shaking for 2 h at −10 °C. The T6P were extracted twice from this organic phase by liquid-liquid extraction with water. The aqueous phase containing MeCN was evaporated to dryness, reconstituted in water and cleaned up further by SPE using Oasis MAX cartridges. The cartridges were preconditioned with MeOH and water; and after sample loading, the cartridges were washed with water and MeOH. Subsequently, the T6P were desorbed with a freshly prepared 2%(*v*/v) formic acid solution in MeOH and then evaporated to dryness. The residue was reconstituted with 200 μL of water/MeOH/MeCN 8/7/85% (v/v/v) before injection. T6P was quantified with hydrophilic-interaction chromatography (HILIC) method coupled to electrospray ionization mass spectrometry (ESI-MS) [27].

### Analysis of glycollate, glyoxylate and 2-OG

Glycollate and glyoxylate were determined according to Xu et al. [28] and the content of 2-OG were measured using α-Ketoglutarate Colorimetric/Fluorometric Assay Kit (BioVision, Catalog #K677, Milpitas, USA) according to the manufacturer’s instructions.

### **Determination of** GO **activity**

The activity of GO was assayed as in Booker et al. [29] with minor modifications. Fresh youngest expanded leaf samples were firstly ground into fine powder in liquid nitrogen; the samples (0.5 g) were then homogenized with Tris-HCl buffer containing 0.01% Triton X-100, 5 mM DTT in an ice bath. After centrifugation (5000 g,15 min) at 4 °C, a 2-ml volume of assay mixture containing 50 mM Tris-HCl buffer (pH 7.8), 0.009% (v/v) Triton X-100, 3.3 mM phenylhydrazine HCl (pH 6.8), 50 μl of plant extract and 5 mM glycollate (neutralized to pH 7.0 with KOH) was used to start the reaction at 25 °C. GO activity was determined by following the formation of glyoxylate phenylhydrazone at 324 nm in the UV-vis spectrophotometer (UV-1700, Nikon) for 2 min after an initial lag phase of 1 min.

### Analysis for Gln, Glu and activities of GS and GOGAT

For determination of Gln and Glu content a Glutamine/Glutamate Determination Kit (Sigma Aldrich, Catalog Number GLN1, St Louis, USA) was used following the manufacturer’s instructions.

For GS (EC 6.3.1.2) and GOGAT (EC 1.4.7.1) assays, youngest fully expanded leaves (0.2 g) snap frozen in liquid nitrogen were homogenized in 2 mL of 50 mM Tris-HCl buffer (pH 7.8), containing 1 mM of EDTA, 15% glycerol, 14 mM of 2-mercaptoethanol and 0.1% Triton-X-100. After centrifugation twice (10,000 g, 10 min) at 4 °C, the supernatant was collected and used for measuring the activities of GS and GOGAT [30]. GS activity was measured by estimating the formation of glutamylhydroxamate at 540 nm after mixing with acidified ferric chloride [31]. Activity of GOGAT was determined by monitoring the oxidation of NADH at 340 nm as described by Tang [32].

### Analysis of NR activity and total nitrogen content

For NR (EC 1.6.6.1) activity analysis, frozen youngest expanded leaves (0.2 g) were homogenized in 2 mL of 25 mM phosphate buffer saline (PBS, pH 8.7) containing 10 mM cysteine and 1 mM EDTA and centrifuged. The resulting supernatant was taken for assaying NR activity under optimized conditions by the diazocoupling method using Griess reagent as described by Sanchez-Rodríguez et al. [33]. Total N content is the sum of inorganic and reduced nitrogen [33], including nitrate, proteins and amino acids, and was determined on the whole tissue sample by the micro-Kjeldahl method after digestion in H_2_SO_4_-H_2_O_2_ [31].

### Determination of NO_3_^−^ and NH_4_^+^ nitrogen

For determination of nitrate content, a Plant Nitrate Assay Kit (Cominbio, Catalog Number ZXTD-1-G, Suzhou, China) was used following the manufacturer’s instructions. Fresh youngest expanded leaf sample (0.1 g, most recently fully expanded) was homogenized in distilled water (1 ml), mixed well using a horizontal shaker and incubated at 90 °C for 30 min. After centrifugation (12,000 g, 15 min) at 25 °C, supernatant (20 μl) was collected and 80 μl of salicylic acid added and incubated for 30 min at 25 °C. NaOH (8% (*w*/*v*), 1900 μl) was added to the mixture. The content of nitrate was analyzed by measuring the absorbance at 540 nm (A540) in a microplate reader.

For determination of NH_4_^+^ content, a Plant Ammonium test kit (Cominbio, Catalog Number ZATD-1-G, Suzhou, China) was used following the manufacturer’s instructions. Fresh youngest fully expanded leaf sample (0.1 g) was homogenized in acetic acid (1 ml) and centrifuged (12,000 g, 10 min) at 25 °C. Tris-HCl (300 μl) and Phenylhydrazine hydrochloride (10 μl) were added to the supernatant (200 μl). After incubation for 5 min in boiling water, glycollic acid (500 μl) was added to the mixture. The content of NH_4_^+^ was analyzed by measuring the absorbance at 580 nm (A580) in a microplate reader.

### Determination of amino acids

The soluble amino acids were extracted from samples (0.2–0.3 g fresh weight) of fully expanded leaves as reported previously [34]. They were derivatized with fluorescence reagent 6-aminoquinolyl-N-hydroxysuccinimidylcarbamat (AQC) (AccQ-FluorTM Reagent Kit; Waters) and then determined with the reverse-phase HPLC System Alliance 2795 (Waters). The chromatographic conditions were as follows: reverse-phase column (XBridge; 150 nm, 5 μm); excitation wavelength, 300 nm; detection wavelength, 400 nm. Eluents consist of buffers A: 140 mM sodium acetate (Merck), 7 mM triethanolamine (Sigma-Aldrich), B: acetonitrile (Roth) and C: purified water (Millipore). The column was equilibrated with buffer A (0.6 ml per min). Separation was performed with gradients buffer B: 1% at 0.5 min, 5% at 27 min, 9% at 28.5 min, 18% at 44.5 min, 60% at 47.5 min and 0% at 50.5 min, at 37 °C column temperature [35]. Chromatograms were analyzed by Waters Empower software (Milford, MA, USA).

### Expression analysis of genes of Tre metabolism

Quantitative real-time PCR (qRT-PCR) was performed in a quantitative PCR instrument (Cat. No. ABI7500, Applied Biosystems Inc., USA). Total RNA was extracted from seedling leaves after treatment for different times as indicated using an RnaExTM Total RNA Isolation Solution (Cat. No. GK3006, GENEray, Shanghai) according to the manufacturer’s instructions. Total RNA (5 μg) was used in reverse transcription with a Rayscript cDNA Synthesis KIT (Cat. No. GK8030, GENEray, Shanghai). qRT-PCR was performed using a AceQ TM qPCR Probe Master Mix (Cat. No. Q112–02, GENEray, Shanghai). PCR used an initial denaturing stage at 95 °C for 4 min with 40 cycles as follows: 95 °C for 10 s, 60 °C for 34 s, and 72 °C for 10 s, followed by 72 °C for 10 min. All quantifications were normalized to the amplification of NtHSP70 gene (locus number DV161835), the relative expression of *TPS1*, *TPS5*, *TPS8*, *TPPA* and *TPPB* were calculated using a comparative △△ threshold cycle method. Primer sequences of all genes used for qRT-PCR are listed in Table 1. Experiments were performed in triplicate for each treatment.

**Table 1:** Primer sequences for qRT-PCR

Annotation	AGI	Sense (5′ to 3′)	Antisense (5′ to 3′)
NtTPS1	FG161474.1	TTCTTGGGGAAGGATGAGGATGTAT	CTTCTCCGAGTTCGATACAGGCCTA
NtTPS5	FG157491.1	TTTTGTGAGGCCAAATCATGATGCC	GGCGTATTGGTAATTCCAAACAAGC
NtTPS8	FG198612.1	ATTGTCGAAGTCAAACCACAAGGTG	AGGTAATACTTGGCTTTGCTTGGCT
NtTPPA	FG173116.1	ATGAGAAGAGTTGGTCAGCTATTGG	AGAACTCAACAGCTTTCCCCTTGTC
NtTPPB	FG143777.1	ACAACAAATTCTGCTTATCCGTACA	TGAATTAGCATACCCTAATGATTCC
NtHSP70–1	DV161835	AGGTGGAGACATGGGTGGTG	TCATTAGGCACACAGATCTCTG

### Measurements of sucrose, glucose and fructose

The contents of sucrose, glucose, fructose and starch were determined by derivatization through trimethyl chlorosilane and hexamethyl disilylamine followed by gas chromatographic detection under FID mode has been established as described in Cai et al. [36]. Briefly, leaf samples that collected after different treatment for 2, 7, 14 and 21 days as indicated were freeze dried and 1.0 g dry weight was ground to a fine powder (0.45 mm). Following extraction for 60 min with 50% (v /v) acetonitrile by shaking. After settling, 6 mL of supernatant were filtered through a 0.45 μm filter. The content of sucrose, glucose and fructose was measured simultaneously using a TRACE TR-5 column (length 60 m × Inner diameter 0. 25 mm, packing particle size 0. 25 μm, Thermo, Waltham, MA, USA).

### Determination of dry weight and leaf area

The whole seedlings were collected and rinsed with deionized water, surface dried and separated into roots, stems and leaves. The area of the fresh leaves was measured with a meter (AM 300, ADC Bio-scientific Ltd., UK). All parts were subsequently dried at 80 °C for 48 h to obtain dry mass. Plant root ratio (%) was calculated as [dry mass (root) / dry mass (root + shoot)] × 100. Measurements were replicated three times with 5 plants per replicate.

### Statistical analysis

All measurements were replicated at least three-fold and means with standard errors of means (SEs) are given. In some Figures the symbols are larger than the SE bars. Data were analyzed with SPSS-17, using one- way analysis of variance and differences in means between treatments were separated by the least significance difference (LSD) test at 0.05 probability.

## Abbreviations

2-OG: 2-oxoglutarate
Ala: Alanine
Arg: Arginine
Asp: Aspartate
Gln: Glutamine
Glu: Glutamate
Gly: Glycine
GO: Glycolate oxidase
GOGAT: Glutamine oxoglutarate aminotransferase
GS: Glutamine synthetase
Leu: Leucine
Met: Methionine
NH_4_^+^: Ammonium
NO_3_^−^: Nitrate
NR: Nitrate reductase
Phe: Phenylalanine
Pn: Net photosynthetic rate
Pro: Proline
Ser: Serine
T6P: Trehalose 6-phosphate
TPP: Trehalose phosphate phosphatase
TPS: Trehalose phosphate synthase
Tre: Trehalose
Tyr: Tyrosine
